# LncRNA AFAP1-AS1 Supresses miR-139-5p and Promotes Cell Proliferation and Chemotherapy Resistance of Non-small Cell Lung Cancer by Competitively Upregulating RRM2

**DOI:** 10.3389/fonc.2019.01103

**Published:** 2019-10-22

**Authors:** Na Huang, Wei Guo, Ke Ren, Wancheng Li, Yi Jiang, Jian Sun, Wenjing Dai, Wei Zhao

**Affiliations:** ^1^Department of Respiratory Medicine, The First Affiliated Hospital of Chengdu Medical College, Chengdu, China; ^2^School of Laboratory Medicine/Sichuan Provincial Engineering Laboratory for Prevention and Control Technology of Veterinary Drug Residue in Animal-origin Food, Chengdu Medical College, Chengdu, China

**Keywords:** AFAP1-AS1, miR-139-5p, RRM2, non-small cell lung cancer, EGFR/AKT

## Abstract

Non-small cell lung cancer (NSCLC) is the leading cause of cancer-related death worldwide. This study aims to understand the underlying mechanism of lncRNA, actin filament-associated protein 1 antisense RNA 1(AFAP1-AS1) in mediating chemotherapeutic resistance in NSCLC. The levels of AFAP1-AS1 in NSCLC tissues and cells were determined using RT-PCR. The protein levels of RRM2, EGFR, and p-AKT were analyzed using Western blotting. Binding between AFAP1-AS1 and miR-139-5p was confirmed using dual luciferase reporter and RNA immunoprecipitation (RIP) assays, and binding between miR-139-5p and RRM2 was confirmed by a dual luciferase reporter assay. NSCLC cell proliferation, apoptosis, and colony formation were examined using MTT, flow cytometry, and colony formation assays, respectively. It was found that AFAP1-AS1 expression was upregulated in NSCLC tissues and cells. In addition, AFAP1-AS1 bound to and downregulated the expression of miR-139-5p, which was reduced in NSCLC tissues. Knockdown of AFAP1-AS1 and overexpression of miR-139-5p inhibited NSCLC cell proliferation, colony formation and chemotherapy resistance and increased cell apoptosis. Additionally, AFAP1-AS1 upregulates RRM2 expression via sponging miR-139-5p. Furthermore, AFAP1-AS1 enhanced NSCLC cell proliferation and chemotherapy resistance through upregulation of RRM2 by inhibiting miR-139-5p expression. Moreover, RRM2 promoted cellular chemotherapy resistance by activating EGFR/AKT. Finally, knockdown of AFAP1-AS1 significantly suppressed tumor growth and chemoresistance in nude mice. In conclusion, AFAP1-AS1 promoted chemotherapy resistance by supressing miR-139-5p expression and promoting RRM2/EGFR/AKT signaling pathway in NSCLC cells.

## Introduction

Lung cancer is the leading cause of cancer-related death worldwide (1–3). Non-small cell lung cancer (NSCLC) accounts for approximately 80% of all lung cancer cases and comprises two histological subtypes, adenocarcinoma (AD) and squamous cell cancer (SCC) (4, 5). The current overall 5-year survival rate for NSCLC is <15% due to both limited therapeutic options and recurrence (4). The prognosis of NSCLC is affected by chemotherapy resistance (6, 7). Thus, a better understanding of carcinogenesis and chemotherapy resistance is critical for developing novel therapies to treat NSCLC patients.

Long non-coding RNAs (lncRNAs) are a family of non-coding RNAs with lengths of >200 nucleotides. Accumulating evidence suggests that lncRNAs contribute to cancer initiation and progression and chemotherapy resistance (8–11). For example, the highly conserved lncRNA MALAT1 is a predictive biomarker for metastasis of lung cancer (12). Elevated LINC00473 expression often correlates with poor prognosis and is a robust biomarker for LKB1-inactivated NSCLC (13). HOTAIR is involved in the invasion and motility of lung cancer cells (14). However, MEG3 serves as a tumor suppressor in NSCLC, inhibiting cell proliferation and inducing p53-mediated cancer cell apoptosis (15).

LncRNA actin filament-associated protein 1 antisense RNA 1 (AFAP1-AS1) is a 6.8-kb lncRNA located on chromosome 4p16.1. AFAP1-AS1 participates in the development of various cancers, including pancreatic ductal adenocarcinoma (16), esophageal adenocarcinoma (17), hepatocellular carcinoma (18), nasopharyngeal carcinoma (19), gallbladder cancer (20), and colorectal cancer (21). In addition, AFAP1-AS1 plays roles in NSCLC tumourigenesis by epigenetically repressing p21 expression (22, 23). However, the molecular mechanisms and global gene regulation mediated by AFAP1-AS1 and the role of AFAP1-AS1 in chemotherapy resistance in human NSCLC has not been explored.

Ribonucleoside-diphosphate reductase subunit M2 (RRM2) is the catalytic subunit of ribonucleotide reductase and modulates the enzymatic activity, which is essential for DNA replication and repair (24). RRM2 has been reported to be involved in the progression of various cancers, including gliomas (25), colorectal cancer (26), bladder cancer (27) and NSCLC (28–31). In addition, RRM2 is a prognostic biomarker for NSCLC (28–31). Interestingly, AKT-induced tamoxifen resistance is reversed by RRM2 inhibition in breast cancer (32), suggesting that RRM2 may participate in the chemotherapy resistance of cancer cells. The abnormal overexpression or activation of AKT has been observed in cancers including lung, ovarian and pancreatic cancers (33), and AKT could be activated by epidermal growth factor receptor (EGFR) (34), implying that targeting EGFR or AKT could offer important approaches for cancer prevention and therapy. Subsequently, we investigated the effect of RRM2 on EGFR/AKT signaling.

In this study, we investigate the role of AFAP1-AS1 in NSCLC cell proliferation and chemotherapy resistance to DDP (Cisplatin) and 5-FU (fluorouracil), which are commonly used for countering progression of cancers in clinic. We also explore the function of RRM2 in the chemotherapy resistance of NSCLC cells. Our data indicate that AFAP1-AS1 expression was elevated in patients with NSCLC and that AFAP1-AS1 acts as a competing endogenous RNA for miR-139-5p, which is an important suppressor in several tumors (35–39). Knockdown of AFAP1-AS1 or overexpression of miR-139-5p inhibited the proliferation, increased the apoptosis, and attenuated the chemotherapy resistance of lung cancer cells by upregulating RRM2. In addition, knockdown of AFAP1-AS1 reduced tumor volume and weight *in vivo*. Taken together, AFAP1-AS1 supresses miR-139-5p and promotes cell proliferation and chemotherapy resistance of NSCLC cells by competitively upregulating RRM2 expression.

## Materials and Methods

### Tissue Collection

This study was approved by the ethics committee of first affiliated hospital of Chengdu Medical College. From Feb. 2018 to Apr. 2019, a total of 44 NSCLC patients were recruited from Department of Respiratory Medicine, the First Affiliated Hospital of Chengdu Medical College Chengdu. All participants signed an informed consent form. NSCLC tissues and adjacent normal lung tissues were collected and stored at −80 °C until used. The drugs cisplatin (DDP), 5-fluorouracil (5-FU), adriamycin, and paclitaxel were used for NSCLC treatment in all patients. In accordance with the Response Evaluation Criteria in Solid Tumors, we grouped the patients with a complete or partial response as responders and defined those with stable or progressive disease as non-responders. The clinicopathological characteristics of the patients with NSCLC are summarized in Table 1.

**Table 1:** Association between lncRNA AFAP1-AS1 expression to clinical factors in the NSCLC tissues.

**Factor**	**AFAP1-AS1 level (High)**	**AFAP1-AS1 level (Low)**	***P*-value (High vs. low)**
**Sex**			0.409
Male	15	13	
Female	9	7	
**Age**			0.556
≤ 60	13	16	
>60	8	7	
**Smoker**?			
Yes	17	15	0.622
No	16	16	
**Histology**			0.437
SSC	17	16	
AC	5	6	
Others	0	0	
**Tissue differentiation**			0.024
Middle and high	18	7	
Low	14	5	
**TNM stage**			0.017
I/II	18	10	
III/IV	11	5	
**Lymph node metastasis**			0.041
Present	15	9	
Absent	13	7	

*SCC, lung squamous cell carcinoma; AC, lung adenocarcinoma; P value < 0.05 was statistically significant*.

### Cell Culture

The NSCLC cell lines H1975, PC-9, A549, and SPCA-1, and a human normal lung epithelial cell line BEAS-2B were purchased from the Institute of Biochemistry and Cell Biology of the Chinese Academy of Sciences (Shanghai, China). The H1975 and SPCA-1 cells were maintained in RPMI 1640 basic medium (GIBCO, Carlsbad, CA), and the PC-9 and A549 cells were cultured in DMEM (GIBCO) in a humidified incubator at 37°C with 5% CO_2_. All media were supplemented with heat-inactivated 10% fetal bovine serum (FBS) and antibiotics (100 U/mL penicillin and 100 mg/mL streptomycin) (GIBCO).

### Cell Transfection

The cells were plated in dishes or plates and grown to 70% confluence for the transfection of small interfering RNA (siRNA) or plasmids using Lipofectamine 2000 (Thermo Fisher Scientific, Shanghai, China). The siAFAP1-AS1#1 and siAFAP1-AS1#2 sequences, the miR-139-5p mimic or inhibitor, the pcDNA-RRM2 plasmid, and the Lv-AFAP1-AS1 knockdown (KD) (Lv-siAFAP1-AS1#1) construct and their paired controls were synthesized by GenePharma (Shanghai, China). The pcDNA-RRM2 plasmid contained the full-length RRM2 coding mRNA sequence (NM_001165931.1), the pcDNA-AFAP1-AS1 plasmid contained the full-length AFAP1-AS1 sequence (ENST00000608442.1), and the Lv-AFAP1-AS1 KD lentivirus expressed siRNA targeting AFAP1-AS1. The siRNA sequences targeting AFAP1-AS1 were designed as follows: siAFAP1-AS1, 5′-GCA TTA TTT TGC TAA TTC AAC-3′ and the scrambled negative control siRNA was the sequence: 5′-CCT AAC CAC AAA CTC TAC GGC-3′ (abbreviated as scramble). The inhibitor sequences targeting miR-139-5p were designed as follows: 5′-CUG GAG ACU GCG ACU GUA GAC UGG AGA CUG CGA CUG UAG ACU GGA GAC UGC GAC UGU AGA CUG GAG ACU GCG ACU GUA GAC UGG AGA CUG CGA CUG UAG A-3′, and the miR-139-5p mimic sequences were designed as follows: 5′-UCU ACA GUG CAC GUG UCU CCA G-3′, and the negative control sequences were 5′-UCU CCG AAC GUG UCA CGU-3′ (abbreviated as pre-NC, or NC). Then, both the full length and mutant of AFAP1-AS1 (or RRM2 3′UTR) were constructed into pmirGLO plasmid for luciferase assay.

### RNA Extraction and Real-Time Polymerase Chain Reaction (PCR) Analyses

Total RNA was extracted from NSCLC tissues or cells with TRIzol reagent (Thermo Fisher Scientific) following the manufacturer's instructions. The expression of AFAP1-AS1, miR-139-5p, and RRM2 was analyzed with SYBR Green Master Mix (Takara, Beijing, China). Complementary DNA (cDNA) was synthesized using a PrimeScript RT Reagent Kit and gDNA Eraser (Takara). Real-time PCR was carried out on an ABI 7500 Real-Time PCR System (Applied Biosystems, Foster City, CA, USA). The primers used were as follows: AFAP1-AS1, 5′-TCG CTC AAT GGA GTG ACG GCA-3′ (forward) and 5′-CGG CTG AGA CCG CTG AGA ACT-3′ (reverse); miR-139-5p, 5′-TCT ACA GTG CAC GTG TCT CCA G-3′ (forward) and 5′-GTG CAG GGT CCG AGG T-3′ (reverse); U6, 5′-TGC GGG TGC TCG CTT CGG CAG C-3′ (forward) and 5′-GTG CAG GGT CCG AGG T-3′ (reverse); and GAPDH, 5′-CAC CCA CTC CTC CAC CTT TG-3′ (forward) and 5′-CCA CCA CCC TGT TGC TGT AG-3′ (reverse); RRM2, 5′-GGC GCG GGA GAT TTA AAG GC-3′ (forward) and 5′-CGG AGG GAG AGC ATA GTG GA-3′ (reverse). The relative expression levels of AFAP1-AS1, miR-139-5p, and RRM2 were calculated using the 2^−ΔΔCt^ method with U6 or GAPDH as the internal control.

### Luciferase Reporter Assay

The luciferase reporter vector pGLO-basic (Promega, Beijing, China) containing the wild-type (WT) or mutant AFAP1-AS1 sequences 1/2 (Mut 1/2) were transfected into A549 cells. The pGLO plasmids containing the full-length RRM2 3′ UTR or its corresponding mutant were co-transfected with an miR-139-5p mimic or inhibitor into A549 cells. After 48 h of incubation, the cells were harvested and luciferase activity was determined using a dual luciferase assay kit (Promega).

### MTT Assay and CCK-8 Assay

Cell proliferation was measured by a 3-(4,5-dimethylthiazol-2-yl)-2,5-diphenyl-tetrazolium bromide (MTT) assay. Lung cancer cells were plated in a 96-well plate (2 × 10^3^ cells/well). After cells were incubated with MTT (Sigma, Shanghai, China), the optical density (OD) value was determined at 450 nm. Additionally, we used a Cell Counting Kit-8 (CCK-8, Sigma) to analyse NSCLC cell viability. In the MTT and CCK-8 assays, the inhibition rate (%) = 100% × (1–OD value of the treated sample/OD value of the control sample).

### Colony Formation Assay

NSCLC cells were seeded in fresh six-well plates in a 5% CO_2_ incubator at 37°C and were then transfected with the indicated siRNAs. Following incubation for 2 weeks, NSCLC cells would grow into colonies, and they were fixed with methanol and stained with 0.1% crystal violet. Visible colonies were manually counted and recorded.

### Apoptosis Analyses

Treated cells were collected, centrifuged at 2,000 rpm for 5 min and washed with PBS three times. The cells were resuspended in 100 μL of PBS, and annexin V/FITC (5 μL) and propidium iodide (PI) (1 μL) were added to each sample. After 15 min incubation at room temperature in the dark, the apoptosis of the cancer cells was analyzed on an S3e flow cytometer (Bio-Rad, Shanghai, China). Cells stained with either annexin V or PI were counted as apoptotic cells.

Caspase-3 activity was also checked in cancer cells by Caspase-3 Activity Assay Kit (Beyotime, Shanghai, China) followed the instruction.

### RNA Immunoprecipitation (RIP) Assays

Rip experiments were performed using a Magna RIP RNA-Binding Protein Immunoprecipitation Kit (Millipore, USA) according to the manufacturer's instructions. Antibodies against EZH2 and AGO-2 were obtained from Sigma. The AGO2 expression level was determined by immunoprecipitation and Western blotting, and the AFAP1-AS1 expression level was determined by real-time PCR.

### Chemical Treatment in Cells

In this study, transfected NSCLC cells were incubated with DDP (solute in PBS) at concentrations of 0, 1, 2, 4, 8, and 12 μM or with 5-FU (solute in PBS) at concentrations of 0, 1, 4, 8, 16, and 32 μM for 36 h. DDP and 5-FU were purchased from Sigma.

A549 and SPCA-1 cells were treated with AST1306 (Selleck, Shanghai, China) at 1 μM for 24 h.

### Generation of Drug-Resistant Cell Lines

The DDP- or 5-FU-resistant cell lines were generated by incubating NSCLC cells with increasing concentration of the indicated drugs. NSCLC cells were plated into plates and maintained in medium containing 0.2 μM DDP. After 48 h incubation, the 0.2 μM DDP-containing medium was discarded, and medium containing gradually increasing concentrations of DDP was added. Finally, cells resistant to 10 μM DDP were obtained and named A549/DDP or SPCA-1/DDP cells.

### Western Blot Analysis

Tissues and cancer cells were lysed using RIPA lysis buffer (Beyotime, Haimen, China) containing protease inhibitor cocktail (Roche). Approximately 20 μg of extracted protein was separated by 10% sodium dodecyl sulfate-polyacrylamide gel electrophoresis (SDS-PAGE) and transferred to 0.22 mm polyvinylidene difluoride (PVDF) membranes (Millipore, Shanghai, China). We blocked the PVDF membranes in 2% bovine serum albumin (BSA) and incubated them with primary antibodies against RRM2 (catalog No. #65939), EGFR (catalog No. #2085), p-AKT (catalog No. #4060), AKT (catalog No. #9272), and GAPDH (catalog No. #5174) (Cell Signaling Technology, Shanghai, China). Then, these immunoblots were incubated with horseradish peroxidase-conjugated secondary antibodies for 60 min at room temperature. GAPDH was used as the internal control.

### Pull Down Assay With Biotinylated AFAP1-AS1 DNA Probe

The biotin-labeled ABHD11-AS1 DNA probe was designed (Thermo), dissolved in binding and washing buffer and mixed with M-280 streptavidin magnetic beads (Thermo) to generate probe-coated beads according to the manufacturer's instruction. The A549 cell lysates were incubated with the probe-coated beads. Then, we used real-time PCR analysis to determine the beads-binding RNAs. The AFAP1-AS1 pull-down probe sequence was 5′-Bio-AGT AAA CAC GCA GTT GCA CAT GGC TGG GGA GGC CTC AGA ATC ATG GCG GGA GGC GAA AGA CAC TTC TTA CGT GGC AGC AGC-3′; and random pull-down probe sequence used as negative control was 5′-Bio-TGC ATC CAA GCC GAT TGC GGT AAC GTG CAT CCA AGC CGA TTG CGG TAA CG-3′.

### Xenograft Tumor Assay

Male athymic nude BALB/c mice were purchased from the Model Animal Research Center of Nanjing University (Nanjing, China). At 5 weeks of age, the mice were randomly divided into four groups. The animal procedure was approved by the Ethics Committee of Animal Experiments of Chengdu Medical College (Chengdu, China). A549 cells were transfected with Lv-scramble or Lv-AFAP1-AS1 KD. 2 × 10^6^ cells were inoculated subcutaneously into the mice. After 10 days, the mice were administered 3 mg/kg (body weight) DDP or PBS every 4 days for 28 days. During this period, the tumor lengths and widths were measured, and tumor volumes were calculated as follows: tumor volume = (length × width^2^)/2. Finally, the tumors were harvested and weighed.

### Bioinformatic Analyses

In this study, LNCipedia version 5.2, lncBase version 2, and starBase were used to predict the potential binding sites between AFAP1-AS1 and miR-139-5p. miRBase and miRDB were applied to analyse the binding sites between miR-139-5p and the RRM2 3′ UTR. In addition, we predicted the RNA-binding activity by examining previous studies (40).

### Statistical Analysis

Statistical analyses were performed using SPSS software (version 19.0). The data are expressed as means ± standard deviations (S.D.). A two-tailed Student's t-test was used to analyse difference between two groups. For multi-group comparisons, we used one-way analysis of variance (ANOVA) with a *post-hoc* Tukey's honestly significant difference (HSD) test. *P*-value of < 0.05 were considered statistically significant.

## Results

### AFAP1-AS1 Is Overexpressed in NSCLC Tissues and Cells

**Figure 1:**
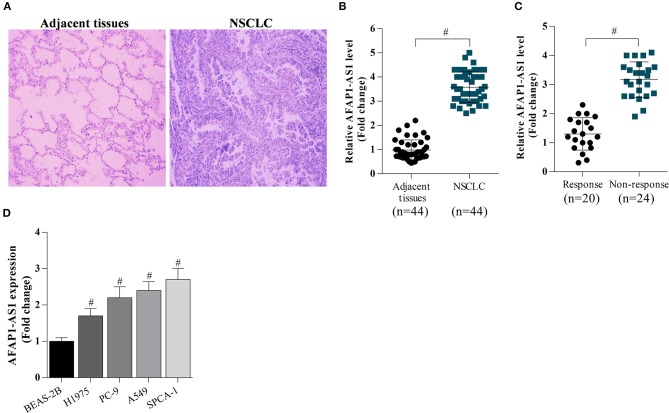
AFAP1-AS1 expression in NSCLC tissues and cells. **(A)** H&E staining of lung clinic tissues. **(B)** RT-PCR on the expression of AFAP1-AS1 in the 44 NSCLC tissues and in 20 adjacent normal tissues. **(C)** RT-PCR on the expression of AFAP1-AS1 in NSCLC tissues of patients in chemotherapy response group (*n* = 20) and the chemotherapy non-response group (*n* = 24). **(D)** AFAP1-AS1 expression in lung cancer cells analyzed by RT- PCR. The results shown as means ± S.D. ^#^*P* < 0.05 compared with BEAS-2B cells.

Firstly, NSCLC tissues and adjacent tissues were collected from hospital. The tissues were analyzed by H&E staining, and the results showed that abnormal cell over-growth appeared in tumors (Figure 1A). RT-PCR was performed to determine the expression of AFAP1-AS1 in NSCLC tissues and cells. It was found that the expression of AFAP1-AS1 was significantly higher in NSCLC tissues than in normal tissues (Figure 1B). In addition, AFAP1-AS1 was overexpressed in NSCLC tissues of patients in the chemotherapy non-response group compared to the chemotherapy response group (Figure 1C). Moreover, Additionally, the expression of AFAP1-AS1 was higher in NSCLC cells than in BEAS-2B cells and was highest in SPCA-1 cells and lowest in H1975 cells (Figure 1D).

### AFAP1-AS1 Inhibits miR-139-5p Expression

**Figure 2:**
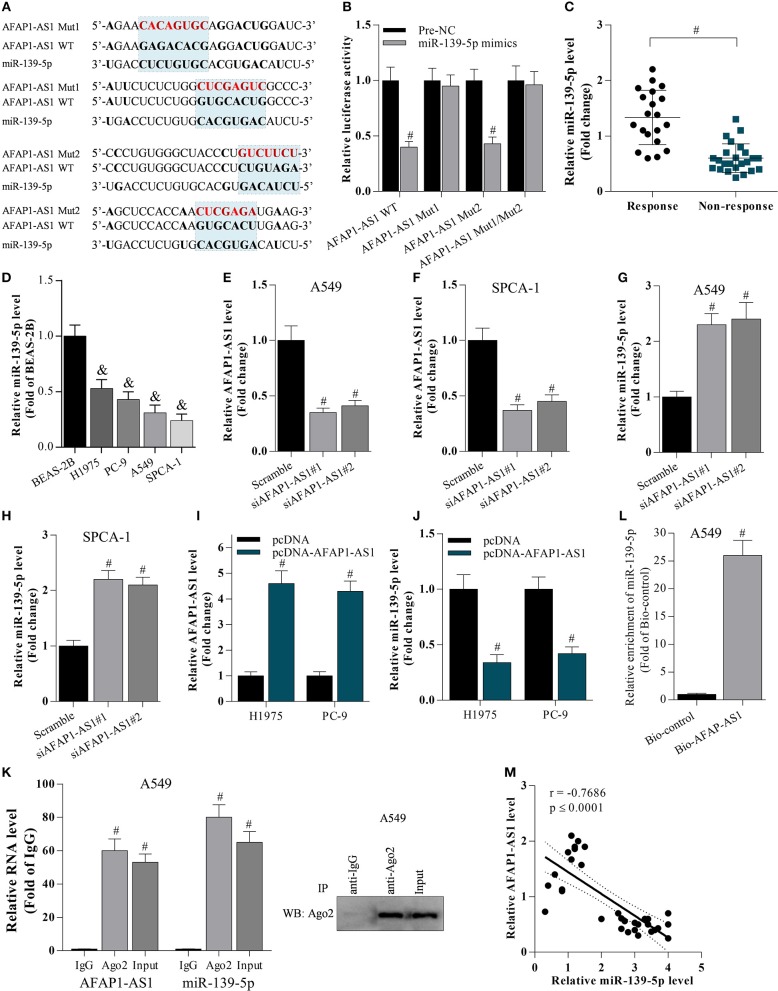
AFAP1-AS1 supresses miR-139-5p expression. **(A)** The potential binding sites between AFAP1-AS1 and miR-139-5p predicted by bioinformatics. “AFAP1-AS1 Mut1” represents the mutation of the first two binding sites, and “AFAP1-AS1 Mut2” represents the mutation of the latter two binding sites. **(B)** A dual luciferase reporter assay on cells transfected with AFAP1-AS1 WT, AFAP1-AS1 Mut1, and AFAP1-AS1 Mut2. Data shown as means ± S.D. ^#^*P* < 0.05 compared with the pre-NC-transfected samples. **(C)** RT-PCR on the miR-139-5p expression in chemoresistant tissues. Data shown as means ± S.D. ^#^*P* < 0.05 compared with chemoresponsive tissues. **(D)** RT-PCR on the miR-139-5p expression in cancer cells. Data shown as means ± S.D. ^&^*P* < 0.05 compared with BEAS-2B cells. **(E–H)** RT-PCR on the effect of AFAP1-AS1 knockdown on miR-139-5p mRNA expression. Data shown as means ± S.D. ^#^*P* < 0.05 compared with the scramble-transfected group. **(I,J)** The effect of AFAP1-AS1 overexpression on miR-139-5p mRNA expression analyzed by RT- PCR. Data shown as means ± S.D. ^#^*P* < 0.05 compared with the pcDNA-transfected group. **(K)** Cell lysate incubated with an anti-Ago2 antibody for RIP, and the AFAP1-AS1 content detected by RT- PCR. Data shown as means ± S.D. ^#^*P* < 0.05 compared with the IgG control group. **(L)** Cell lysate incubated with Bio-AFAP1-AS1 for RIP, and the enrichment of miR-139-5p detected by RT- PCR. Data shown as means ± S.D. ^#^*P* < 0.05 compared with Bio-control group. **(M)** The expression of AFAP1-AS1 and miR-139-5p negatively correlated in NSCLC tissues. *r* = −0.7686 and *p* ≤ 0.0001.

The potential binding sites between AFAP1-AS1 and miR-139-5p were predicted based on bioinformatic analysis (Figure 2A). The dual luciferase reporter assay demonstrated that the miR-139-5p mimic significantly reduced the luciferase activity of cells transfected with AFAP1-AS1 WT as well as that of cells transfected with the AFAP1-AS1 mutated type AFAP1-AS1 Mut2 (Figure 2B). However, the miR-139-5p mimic failed to suppress the luciferase activity of cells transfected with the other AFAP1-AS1 mutated type Mut1, suggesting that miR-139-5p may bind to more than one site on the AFAP1-AS1 Mut1 construct (Figure 2B). We found that the level of miR-139-5p was lower in patients in the chemotherapy non-response group than in the chemotherapy response group (Figure 2C), and miR-139-5p was decreased in lung cancer cell lines compared with BEAS-2B cells (Figure 2D). Furthermore, transfection with siRNA targeting AFAP1-AS1 reduced AFAP1-AS1 expression (Figures 2E,F) and upregulated miR-139-5p expression (Figures 2G,H) in A549 and SPCA-1 cells. In contrast, pcDNA-AFAP1-AS1-mediated overexpression of AFAP1-AS1 reduced the miR-139-5p level in H1975 and PC-9 cells (Figures 2I,J). AFAP1-AS1 expression was significantly elevated in anti-Ago2 (Protein argonaute-2)-incubated A549 cells (Figure 2K), and AFAP1-AS1 could directly bind to miR-139-5p (Figure 2L). There was a negative correlation between AFAP1-AS1 and miR-139-5p expression in NSCLC cells (Figure 2M). These findings indicated that AFAP-AS1 was a sponge of miR-139-5p.

### Suppression of AFAP1-AS1 or Overexpression of miR-139-5p Inhibits the Proliferation and Increases Cell Apoptosis of NSCLC Cells

**Figure 3:**
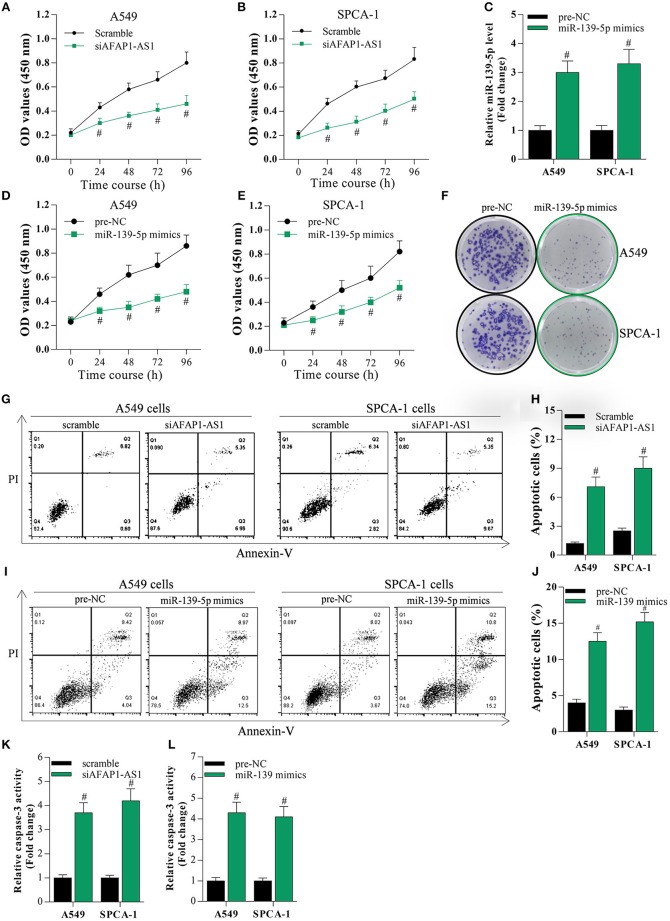
Suppression of AFAP1-AS1 or overexpression of miR-139-5p inhibits the proliferation and increases cell apoptosis of NSCLC cells. **(A,B)** MTT assay on the proliferation of A549 and SPCA-1 cells with AFAP1-AS1 knockdown. Data shown as means ± S.D. ^#^*P* < 0.05 compared with the scramble-transfected cells. **(C)** MiR-139-5p overexpressed in A549 and SPCA-1 cells. Data shown as means ± S.D. ^#^*P* < 0.05 compared with the pre-NC-transfected group. **(D,E)** MTT assay on the proliferation of A549 and SPCA-1 cells with miR-139-5p overexpression. Data shown as means ± S.D. ^#^*P* < 0.05 compared with the pre-NC-transfected group. **(F)** A colony formation assay on the effect of miR-139-5p overexpression on cell proliferation. **(G,H)** Annexin V/PI staining to assess the effect of AFAP1-AS1 knockdown on the apoptosis of A549 and SPCA-1 cells. The cell apoptosis data shown as means ± S.D. ^#^*P* < 0.05 compared with the scramble-transfected cells. **(I,J)** Annexin V/PI staining to assess the effect of miR-139-5p overexpression on the apoptosis of A549 and SPCA-1 cells. **(K,L)** Caspase-3 activity was identified in A549 and SPCA-1 cells, which were treated as indicated. The cell apoptosis data shown as means ± S.D. ^#^*P* < 0.05 compared with the pre-NC-transfected cells.

To investigate the effect of AFAP1-AS1 and miR-139-5p on the proliferation and apoptosis of NSCLC cells, A549 and SPCA-1 cells were transfected with scramble, siAFAP1-AS1, pre-NC, or the miR-139-5p mimic. Knockdown of AFAP1-AS1 suppressed cancer cell proliferation, as evidenced by the MTT assay results (Figures 3A,B). Similarly, overexpression of miR-139-5p decreased cell proliferation (Figures 3C–E) and inhibited colony formation (Figure 3F). As expected, suppression of AFAP1-AS1 or overexpression of miR-139-5p significantly increased the apoptosis of A549 and SPCA-1 cells (Figures 3G–L).

### Knockdown of AFAP1-AS1 or Overexpression of miR-139-5p Decreases the Chemotherapy Resistance of NSCLC Cells

**Figure 4:**
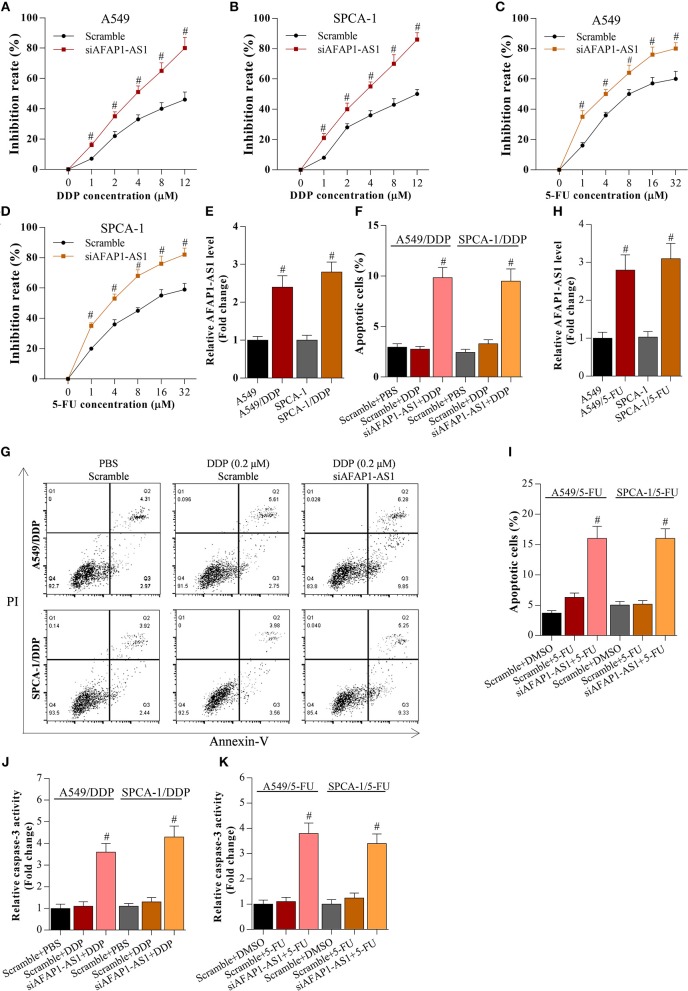
Knockdown of AFAP1-AS1 decreases the chemotherapy resistance of NSCLC cells. **(A,B)** CCK-8 assay on A549 or SPCA-1 cells transfected with scramble or siAFAP1-AS1 and incubated with DDP for 36 h at the indicated concentrations. Data shown as means ± S.D. ^#^*P* < 0.05 compared with the scramble-transfected cells. **(C,D)** CCK-8 assay on A549 or SPCA-1 cells transfected with scramble or siAFAP1-AS1 and incubated with 5-FU for 36 h at the indicated concentrations. Data shown as means ± S.D. ^#^*P* < 0.05 compared with the scramble-transfected cells. **(E)** AFAP1-AS1 levels determined by RT-PCR in A549 and drug-resistant A549/DDP cells as well as in SPCA-1 and SPCA-1/DDP cells. Data shown as means ± S.D. ^#^*P* < 0.05 compared with parental A549 cells. **(F,G)** Knockdown of AFAP1-AS1 increased the DDP-induced apoptosis of drug-resistant NSCLC cells. Data presented as means ± S.D. ^#^*P* < 0.05 compared with the scramble-transfected DDP-resistant cancer cells. **(H)** AFAP1-AS1 levels determined by RT-PCR in A549 and drug-resistant A549/5-FU cells as well as in SPCA-1 and SPCA-1/5-FU cells. Data shown as means ± S.D. ^#^*P* < 0.05 compared with parental A549 cells. **(I)** Knockdown of AFAP1-AS1 increased the 5-FU-induced apoptosis of drug-resistant NSCLC cells. Data presented as means ± S.D. ^#^*P* < 0.05 compared with the scramble-transfected 5-FU-resistant cancer cells. **(J,K)** Caspase-3 activity was identified in A549 and SPCA-1 cells, which were treated as indicated. ^#^*P* < 0.05 compared with the scramble+PBS group.

To analyse the effect of AFAP1-AS1 and miR-139-5p on the chemoresistance of NSCLC cells, scramble- or siAFAP1-AS1-transfected A549 and SPCA-1 cells were incubated with DDP or 5-FU. It was found that the drug-induced growth inhibition increased in a dose-dependent manner, and AFAP1-AS1 knockdown increased the inhibitory activity of DDP and 5-FU in NSCLC cells (Figures 4A–D), implying that suppression of AFAP1-AS1 alleviated the chemotherapy resistance of NSCLC cells. In addition, we also observed that AFAP1-AS1 was significantly overexpressed in DDP-resistant A549 and SPCA-1 cells compared with canonical A549 and SPCA-1 cells (Figure 4E). Furthermore, interfering with AFAP1-AS1 expression significantly increased the DDP-induced apoptosis in the drug-resistant cancer cells (Figures 4F,G). Similarly, AFAP1-AS1 was obviously increased in 5-FU-resistant A549 and SPCA-1 cells, and knockdown of AFAP1-AS1 also promoted 5-FU-triggered cell apoptosis (Figures 4H–K).

**Figure 5:**
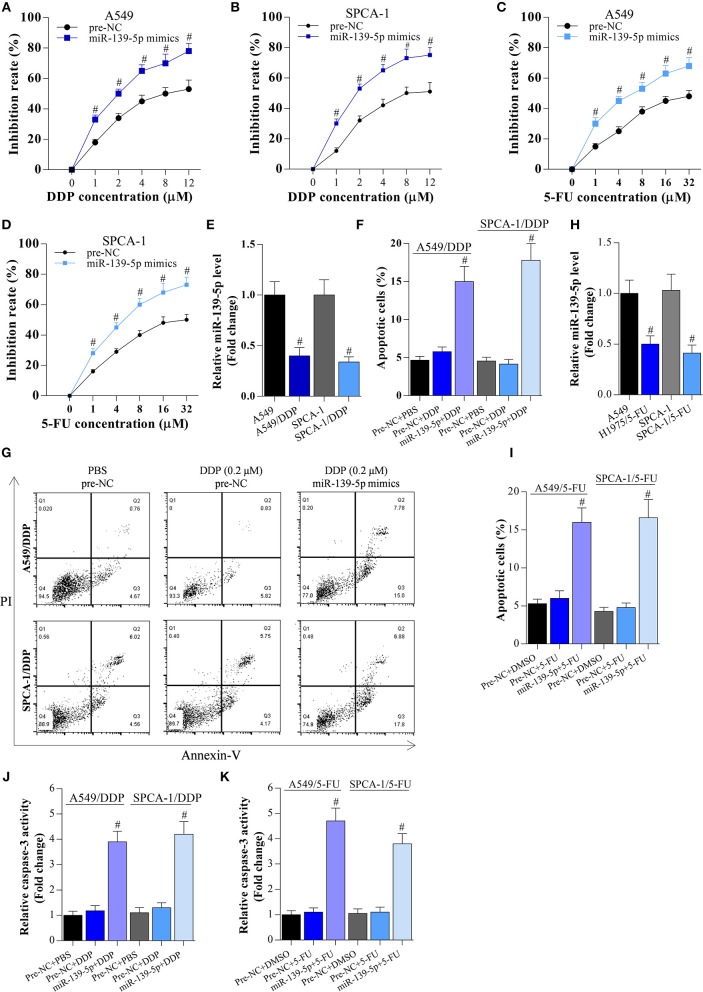
Overexpression of miR-139-5p decreases the chemotherapy resistance of NSCLC cells. **(A,B)** CCK-8 assay on A549 or SPCA-1 cells transfected with pre-NC or miR-139-5p mimic and incubated with DDP for 36 h at the indicated concentrations. Data shown as means ± S.D. ^#^*P* < 0.05 compared with the pre-NC-transfected cells. **(C,D)** CCK-8 assay on A549 or SPCA-1 cells transfected with pre-NC or the miR-139-5p mimic and incubated with 5-FU for 36 h at the indicated concentrations. Data shown as means ± S.D. ^#^*P* < 0.05 compared with the pre-NC-transfected cells. **(E)** RT-PCR on the expression of miR-139-5p in A549 and drug-resistant A549/DDP cells, as well as in SPCA-1 and SPCA-1/DDP cells. Data presented as means ± S.D. ^#^*P* < 0.05 compared with parental A549 cells. **(F,G)** Overexpression of miR-139-5p increased DDP-induced apoptosis in drug-resistant NSCLC cells. Data shown as means ± S.D. ^#^*P* < 0.05 compared with the pre-NC-transfected DDP-resistant cancer cells. **(H)** RT-PCR on the expression of miR-139-5p in A549 and drug-resistant A549/5-FU cells, as well as in SPCA-1 and SPCA-1/5-FU cells. Data presented as means ± S.D. ^#^*P* < 0.05 compared with parental A549 cells. **(I)** Overexpression of miR-139-5p increased 5-FU-induced apoptosis in drug-resistant NSCLC cells. Data shown as means ± S.D. ^#^*P* < 0.05 compared with the pre-NC-transfected 5-FU-resistant cancer cells. **(J,K)** Caspase-3 activity was identified in A549 and SPCA-1 cells, which were treated as indicated. ^#^*P* < 0.05 compared with the scramble+PBS group.

Meanwhile, miR-139-5p mimic enhanced the inhibitory activity of DDP and 5-FU on these cancer cells (Figures 5A–D), indicating that overexpression of miR-139-5p decreased the chemotherapy resistance of NSCLC cells to DDP and 5-FU. Although miR-139-5p expression was reduced in DDP- or 5-FU-resistant NSCLC cells (Figures 5E–H), overexpression of miR-139-5p significantly enhanced the apoptosis of DDP- or 5-FU-resistant cancer cells (Figures 5F–K).

### Cooperation of miR-139-5p and AFAP1-AS1 Regulates RRM2 Expression by Targeting Its 3′ UTR

**Figure 6:**
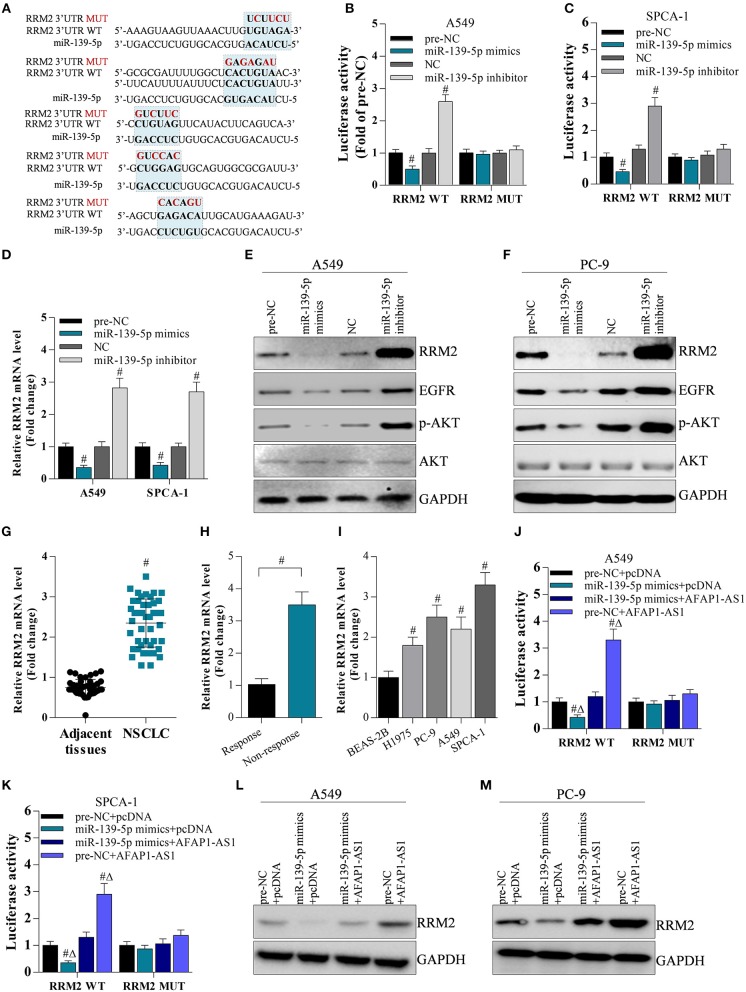
AFAP1-AS1 upregulates RRM2 expression by sponging miR-139-5p. **(A)** The potential binding sites between RRM2 and miR-139-5p predicted by bioinformatics. All binding sites in the RRM2 3′ UTR were mutated and named “RRM2 MUT”. **(B,C)** A dual luciferase reporter assay on A549 cells and SPCA-1 cells with WT RRM2 3′ UTR or mutated 3′ UTR. Data shown as means ± S.D. ^#^*P* < 0.05 compared with the pre-NC-transfected or NC-transfected cancer cells. **(D)** RT-PCR on the cDNA from A549 cells to determine the effect of miR-139-5p on RRM2 mRNA expression. Data shown as means ± S.D. ^#^*P* < 0.05 compared with the pre-NC-transfected or NC-transfected A549 cells. **(E,F)** Western blot on the effect of miR-139-5p on protein expression of RRM2, EGFR, AKT, and p-AKT in A549 and SPCA-1 cells. **(G)** RT-PCR on the expression of RRM2 mRNA in NSCLC tissues (*n* = 44) and in adjacent normal tissues (*n* = 20). Data shown as means ± S.D. ^#^*P* < 0.05 compared with the normal tissues. **(H)** The mRNA level of RRM2 in chemotherapy response (*n* = 4) and resistance (*n* = 7) NSCLC tumors by RT-PCR. The results shown as means ± S.D. ^#^*P* < 0.05 compared with the response group. **(I)** RRM2 mRNA expression in lung cancer cells analyzed by RT- PCR. The results shown as means ± S.D. ^#^*P* < 0.05 compared with BEAS-2B cells. **(J,K)** A dual luciferase reporter assay on A549 cells and SPCA-1 cells with WT RRM2 3′ UTR or mutated 3′ UTR. Data shown as means ± S.D. ^#^*P* < 0.05 compared with the pre-NC-transfected or NC-transfected cancer cells. **(L,M)** Western blot on the effect of miR-139-5p and AFAP1-AS1 on protein expression of RRM2in A549 and SPCA-1 cells.

To investigate the interaction between miR-139-5p and RRM2, a luciferase reporter gene assay was performed. The binding sites between miR-139-5p and the RRM2 3′ UTR were predicted by bioinformatics (Figure 6A). The RRM2 3′ UTR sequences were sub-cloned into the pGLO plasmid, and A549 and SPCA-1 cells were co-transfected with the RRM2 3′ UTR plasmid and the miR-139-5p mimic or inhibitor. The results showed that the miR-139-5p mimic or inhibitor significantly decreased or increased, respectively, the luciferase activity driven by RRM2 WT; however, the miR-139-5p mimic or inhibitor did not affect the luciferase activity driven by the mutated RRM2 3′ UTR (termed RRM2 MUT) (Figures 6B,C). The miR-139-5p mimic or inhibitor noticeably decreased or increased RRM2 mRNA expression (Figure 6D) and protein expression (Figures 6E,F), respectively. Interestingly, the miR-139-5p mimic or inhibitor also modulated the protein levels of EGFR and p-AKT (Figures 6E,F). RRM2 was found to be overexpressed in NSCLC tissues (Figure 6F) and multi-drug resistant NSCLC cells (Figures 6G,H). RRM2 was also found has higher mRNA level in NSCLC cells than normal. To determine the cooperation of miR-139-5p and AFAP1-AS1 on regulation of RRM2 expression, the data showed that AFAP1-AS1 significantly increased luciferase activity of RRM2 3′UTR, and elevated RRM2 protein level (Figures 6J–M). However, AFAP1-AS1-induced elevation of RRM2 could be reversed by overexpression of miR-139-5p in A549 and SPCA-1 cells (Figures 6J–M).

### Knockdown of AFAP1-AS1 Inhibits Cell Proliferation and Alleviates Chemotherapy Resistance Via the miR-139-5p/RRM2 Axis

**Figure 7:**
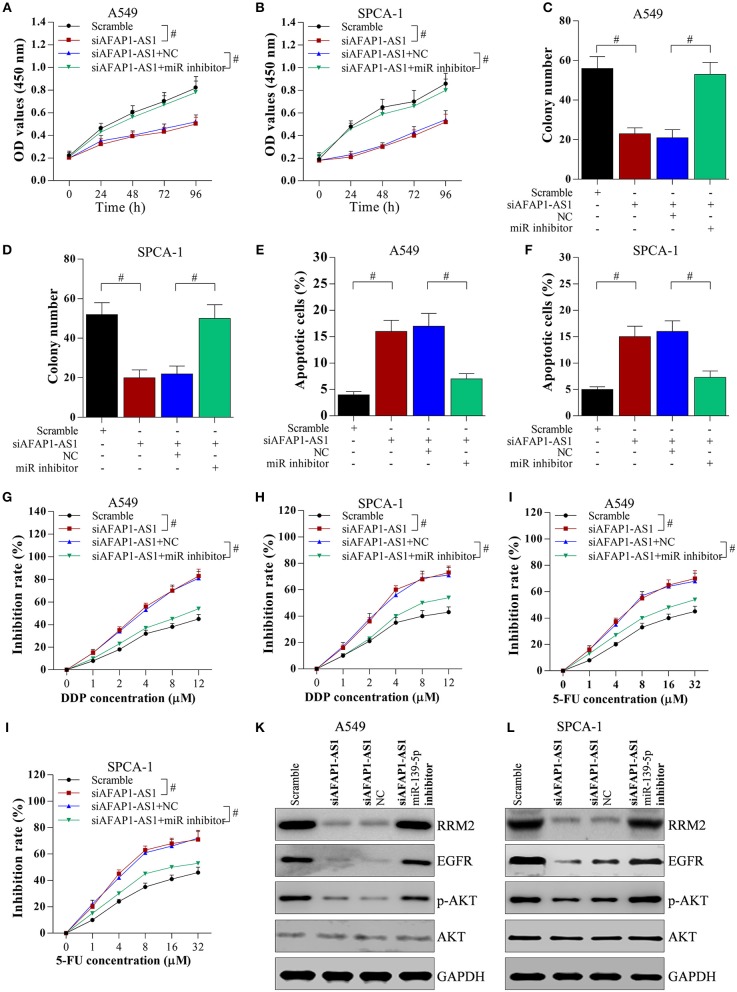
AFAP1-AS1 promotes cell proliferation and chemotherapy resistance through the miR-139-5p/RRM2 axis. A549 or SPCA-1 cells were transfected with scramble, siAFAP1-AS1, siAFAP1-AS1+NC, and siAFAP1-AS1+miR-139-5p inhibitor. **(A,B)** MTT assay showed miR-139-5p inhibitor reversed the inhibitory effect of AFAP1-AS1 knockdown on lung cancer cell proliferation. **(C)** and **(D)** The miR-139-5p inhibitor reversed the inhibitory effect of AFAP1-AS1 knockdown on colony formation. **(E,F)** The miR-139-5p inhibitor reversed the inhibitory effect of AFAP1-AS1 knockdown on apoptosis of NSCLC cells. **(G,H)** In A549 and SPCA-1 cells incubated with DDP for 36 h, the miR-139-5p inhibitor reversed the inhibitory effect of siAFAP1-AS1 on chemotherapy resistance (DDP). **(I,J)** the miR-139-5p inhibitor reversed the inhibitory effect of siAFAP1-AS1 on chemotherapy resistance in cells incubated with 5-FU. **(K,L)**Knockdown of AFAP1-AS1 downregulated RRM2 protein expression and reduced the protein levels of EGFR and p-AKT, while miR-139-5p reversed these effects in A549, and SPCA-1 cells. All data shown as means ± S.D. ^#^*P* < 0.05 indicates a significant difference between the two indicated groups.

To determine the role of the miR-139-5p/RRM2 axis in AFAP1-AS1-mediated cell proliferation and chemotherapy resistance, NSCLC cells were transfected with scramble, siAFAP1-AS1, siAFAP1-AS1+NC, and siAFAP1-AS1+miR-139-5p inhibitor. The miR-139-5p inhibitor reversed the suppressive effect of siAFAP1-AS1 on cell proliferation (Figures 7A,B) and colony formation (Figures 7C,D) and reversed the increased cancer cell apoptosis (Figures 7E,F). In addition, the miR-139-5p inhibitor reversed the suppressive effect of siAFAP1-AS1 on DDP resistance (Figures 7G,H) and 5-FU resistance (Figures 7I,J). Additionally, AFAP1-AS1 knockdown downregulated the protein expression of RRM2 and decreased the protein levels of EGFR and p-AKT. However, the miR-139-5p inhibitor reversed these effects (Figures 7K,L).

### EGFR/AKT Signaling Is Involved in RRM2-Mediated Chemotherapy Resistance

**Figure 8:**
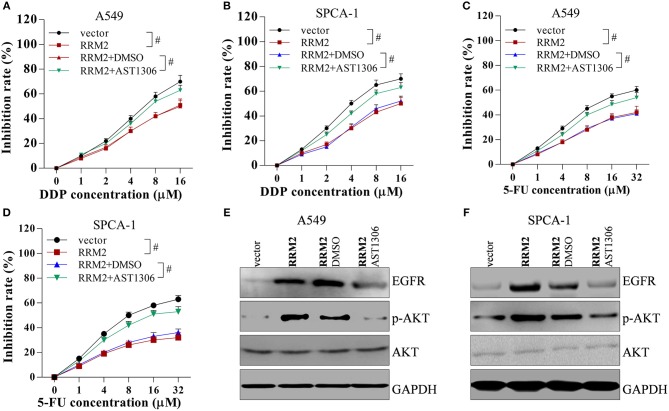
RRM2 increases the chemotherapy resistance of NSCLC cells via EGFR/AKT signaling pathway. Cancer cells were transfected with empty vector, RRM2, RRM2+DMSO and RRM2+AST1306, and then incubated with the EGFR inhibitor AST1306 at 1 μM for 24 h. **(A,B)** In NSCLC cells incubated with DDP for 36 h, overexpression of RRM2 enhanced chemotherapy resistance, and AST1306 reversed this effect. **(C,D)** In NSCLC cells incubated with 5-FU for 36 h, overexpression of RRM2 enhanced chemotherapy resistance and AST1306 reversed this effect. **(E,F)** In A549 and SPCA-1 cells, overexpression of RRM2 elevated the protein expression of EGFR and the level of p-AKT, and AST1306 reversed this effect. All data shown as means ± S.D. ^#^*P* < 0.05 indicates a significant difference between the two indicated groups.

To investigate the role of EGFR/AKT in RRM2-mediated chemotherapy resistance, A549 and SPCA-1 cells were transfected with the pcDNA vector, pcDNA-RRM2, pcDNA-RRM2+DMSO, and pcDNA-RRM2+AST1306 (inhibitor of EGFR). AST1306 reversed the RRM2-mediated promotion of chemotherapy resistance (Figures 8A–D). Additionally, AST1306 reversed the RRM2-induced upregulation of EGFR expression and p-AKT levels in A549 and SPCA-1 cells (Figures 8E,F). These results indicated that RRM2 enhanced the chemotherapy resistance of NSCLC cells via EGFR/AKT signaling pathway.

**Figure 9:**
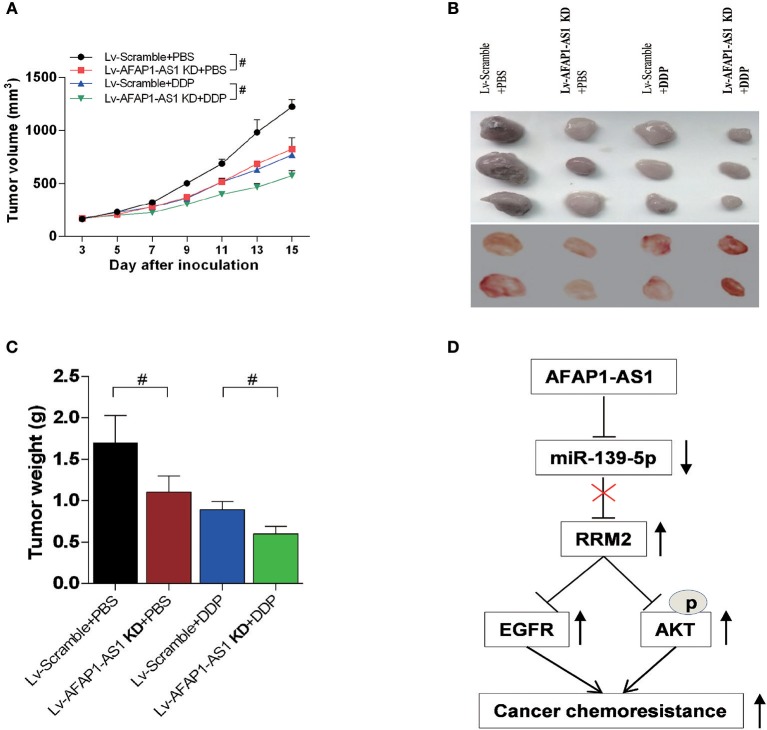
Knockdown of AFAP1-AS1 decreases the tumourigenicity and chemoresistance of NSCLC cells in nude mice. Male BALB/c nude mice injected with A549 cells and treated for 4 weeks as follows: Lv-Scramble+PBS, Lv-AFAP1-AS1 KD+PBS, Lv-Scramble+DDP, and Lv-AFAP1-AS1 KD+DDP. **(A–C)** Tumor volumes, sizes and weights of 2 independent repeats. Data shown as means ± S.D. *n* = 5. ^#^*P* < 0.05 indicates a significant difference between the two indicated groups. **(D)** Schema of the signaling pathways involved in AFAP1-AS1/miR-139-5p/RRM2-mediated chemotherapy resistance in NSCLC cells.

Moreover, the *in vivo* experiments show that knockdown of AFAP1-AS1 suppresses tumorigenicity and chemo-resistance of NSCLC cells in the nude mice (Figures 9A–D). These results indicated that AFAP1-AS1/miR-139-5p/RRM2/EGFR/AKT signaling pathway was involved in the progression of NSCLC (Figure 9D).

## Discussion

LncRNAs are involved in many aspects of cancer development and chemotherapy resistance (8–11). To investigate whether there is an abnormal expression of lncRNA AFAP1-AS1 in NSCLC tissues and cancer cells, real-time PCR was performed. We assessed the effect of AFAP1-AS1 on the proliferation, apoptosis and chemotherapy resistance of lung cancer cells. AFAP1-AS1 could perform as a sponge of miR-139-5p in cancer progression. Suppression of AFAP1-AS1 or overexpression of miR-139-5p significantly repressed the proliferation, increased the apoptosis, and ameliorated the chemotherapy resistance of NSCLC cells by downregulating RRM2. Furthermore, downregulation of AFAP1-AS1 decreased xenograft tumor volume and weight. These findings suggested that AFAP1-AS1 could be an oncogene and induce chemotherapy resistance by modulating miR-139-5p/RRM2 signaling in NSCLC.

Accumulating evidence demonstrated that dysregulated lncRNAs are major contributors to tumourigenesis and cancer development. For example, HOTAIR plays an important role in cellular proliferation, invasion, and clinical relapse in small cell lung cancer (41). HOTAIR also mediates chemoresistance in NSCLC by regulating HOXA1 methylation and could be a potential target for new adjuvant therapy against chemoresistance (42). In addition, the p53-regulated lncRNA TUG1 affects NSCLC cell proliferation in part by epigenetically controlling HOXB7 expression (43). MEG3 acts as a tumor suppressor in NSCLC cell proliferation and induces p53-mediated cancer cell apoptosis (15). Moreover, downregulation of AFAP1-AS1 results in growth inhibition and apoptosis promotion in lung adenocarcinoma cells, indicating that this lncRNA participates in tumourigenesis (44). In NSCLC, AFAP1-AS1 increases tumourigenesis by epigenetically repressing p21 expression. AFAP1-AS1 recruits EZH2 to the p21 promoter region, resulting in downregulation of p21, which is a tumor suppressor (22, 23). Consistent with these previous findings, we found that AFAP1-AS1 was upregulated in NSCLC tissues and cells, and it was overexpressed in chemotherapy-resistant tissues, indicating that AFAP1-AS1 is a positive regulator of NSCLC development and chemoresistance. Previous studies have shown that lncRNAs can act as competing endogenous RNAs of miRNAs (45). Thus, in the present study, we predicted binding sites between AFAP1-AS1 and miR-139-5p, which is a tumor suppressor in colorectal cancer and endometrial cancer (38, 46). However, the role of miR-139-5p in NSCLC has not been explored. Luciferase reporter assays, RIP assays, and real-time PCR were performed, and the data showed that AFAP1-AS1, as a sponge, directly bound to miR-139-5p, leading to downregulation of miR-139-5p expression, and that miR-139-5p expression was decreased in chemotherapy-resistant tissues. We also found that the miR-139-5p inhibitor reversed AFAP1-AS1-induced biological effects, indicating that the interaction of AFAP1-AS1 and miR-139-5p is involved in NSCLC progression and chemotherapy resistance.

In addition, we found that AFAP1-AS1 participated in positively modulating luciferase activity of RRM2 3′ UTR and RRM2 level by acting as a sponge of miR-139-5p in cancer cells, suggesting that RRM2, as AFAP1-AS1, is a oncogenic regulator. This finding is in consistent with previous reports that RRM2 is an oncogene in certain cancers (25–32). It was reported that silencing RRM2 suppresses glioblastoma cell invasion and migration by reducing the expression of metalloproteinase-2 (MMP-2) and MMP-9 (25). The abnormal overexpression or activation of AKT has been observed in many cancers, including lung, ovarian, and pancreatic cancers, and is associated with increased cancer cell proliferation and survival (33). AKT could be activated by epidermal growth factor receptor (EGFR) (34). Consequently, targeting EGFR or AKT could offer important approaches for cancer prevention and therapy. More importantly, overexpression of RRM2 in gastric cancer cells promotes their invasiveness by regulating the AKT/NF-κB signaling pathway (47), and RRM2 increases tumor angiogenesis and growth by modulating the expression of thrombospondin-1 (TSP-1) and vascular endothelial growth factor (VEGF) (48). Subsequently, we investigated the effect of AFAP1-AS1/miR-139-5p/RRM2 signaling on EGFR expression and phosphorylation of AKT. Expectedly, our data showed that overexpression of RRM2 promoted the proliferation, inhibited the apoptosis, and increased the chemotherapy resistance of NSCLC cells through upregulating EGFR expression and AKT phosphorylation. The EGFR inhibitor AST1306 reversed the RRM2-induced effects on cancer cells, indicating that the function of RRM2 is associated with EGFR/AKT signaling pathway. These findings suggested that miR-139-5p inhibited the proliferation and promoted the apoptosis of NSCLC cells by upregulating RRM2/EGFR/AKT signaling pathway. The mutation of EGFR was thought main driver in NSCLC (49, 50), our data shown the mutation of EGFR in H1975 (L858R+T790M) and PC-9 (del19) has little affect on AFAP1-AS1. The underlying mechanism needs more research to lighten.

Taken together, our study demonstrates that AFAP1-AS1 expression is upregulated and miR-139-5p expression downregulated in NSCLC tissues and cells. AFAP1-AS1 promotes NSCLC development and increased chemotherapy resistance by modulating miR-139-5p/RRM2/EGFR/AKT pathway. Suppression of AFAP1-AS1 expression reduced tumor growth and attenuated chemotherapy resistance *in vivo*. Therefore, AFAP1-AS1 could be a promising and therapeutic target of NSCLC.

## Data Availability Statement

All datasets generated for this study are included in the manuscript/supplementary files.

## Ethics Statement

The studies involving human participants were reviewed and approved by the ethics committee of first affiliated hospital of Chengdu Medical College. The patients/participants provided their written informed consent to participate in this study. The animal study was reviewed and approved by the Ethics Committee of Animal Experiments of Chengdu Medical College.

## Author Contributions

WG, KR, and NH performed the experiments, analyzed the data, and prepared the manuscript draft. YJ provided the human specimens. YJ, JS, and WD set up the experiments and repeated the key experiments. WZ and WL conceived the work, analyzed the data, and prepared the manuscript. All authors critically revised the manuscript, approved the final version, and agreed to be accountable for all aspects of the manuscript.

### Conflict of Interest

The authors declare that the research was conducted in the absence of any commercial or financial relationships that could be construed as a potential conflict of interest.
